# Value of CT spectral imaging in the differential diagnosis of sarcoidosis and Hodgkin’s lymphoma based on mediastinal enlarged lymph node: A STARD compliant article

**DOI:** 10.1097/MD.0000000000031502

**Published:** 2022-11-25

**Authors:** Lixiu Cao, Huijing Wu, Yongliang Liu

**Affiliations:** a Department of ECT, Tangshan People’s Hospital, Tangshan, Hebei Province, China; b Department of Neurosurgery, Tangshan People’s Hospital, Tangshan, Hebei Province, China.

**Keywords:** Hodgkin’s lymphoma, sarcoidosis, spectrum CT imaging

## Abstract

To investigate the imaging characteristics of sarcoidosis and Hodgkin’s lymphoma based on mediastinal enlarged lymph node using spectral CT and evaluate whether the quantitative information can improve the differential diagnosis of these diseases. This retrospective study was approved by the institutional review board, and written informed consent was obtained from all patients. Overall, 21 patients with sarcoidosis and 39 patients with Hodgkin’s lymphoma were examined with CT spectral imaging during the arterial phase (AP) and venous phase (VP). The CT values on 40 to 140 keV monochromatic images and iodine (water) concentrations of enlarged lymph nodes were obtained in AP and VP. Iodine concentrations (ICs) were normalized to the iodine concentration in the aorta. The differences in normalized iodine concentrations (NICs) and hounsfield units (HU) curve slop (λ_HU_) were calculated. Anatomical distribution of mediastinal lymph nodes and morphologic features were also compared. Receiver operating characteristic curves were generated to help establish threshold values for the parameters required for the significant differentiation of sarcoidosis from lymphomas. The CT values on 40 to 100 keV monochromatic images in AP and 40 to 50 keV in VP were higher in sarcoidosis than those in Hodgkin’s lymphoma, the differences were statistically significant (*P* < .05); NICs during the AP and λ_HU_ during the AP (VP) in patients with sarcoidosis differed significantly from those in patients with Hodgkin’s lymphoma. Receiver operating characteristic curves analysis showed that the monochromatic CT value on 40 keV in AP had the highest sensitivity (71.4%) and specificity (100%) in differentiating sarcoidosis from Hodgkin’s lymphoma. The anatomic distribution, coalescence, calcification, compression, enhancement pattern and enhancement degree of the mediastinal enlarged lymph node differed significantly between the groups (*P* < .05). The combination of monochromatic CT value, NICs and λ_HU_ had higher sensitivity and specificity than did those of conventional qualitative CT image analysis during the combined phases. CT spectral imaging has promising potential for the diagnostic differentiation of Hodgkin’s lymphomas and sarcoidosis. The monochromatic CT value, iodine content and λ_HU_ could be valuable parameters for differentiating Hodgkin’s lymphomas and sarcoidosis based on mediastinal enlarged lymph node.

## 1. Introduction

Mediastinal enlarged lymph node can occur from a wide range of pathologies. Common malignant conditions include metastatic lymph nodes and malignant lymphomas, for example, Hodgkin’s and non-Hodgkin’s disease. Benign causes include sarcoidosis and granulomatous infectious conditions including tuberculosis, coccidioidomycosis, histoplasmosis and others.^[1]^ Among them, it is extremely important for clinicians and radiologists to be able to distinguish metastatic lymph nodes, lymphoma and sarcoidosis in a clinical setting for effective disease management and accurate prognosis.^[2–4]^ Different from sarcoidosis and lymphoma, mediastinal metastatic lymph nodes are common in middle-aged and elderly patients with primary malignant tumors; In addition, the imaging features of metastatic lymph nodes often show asymmetric distribution and heterogeneity, especially uneven enhancement. Hence, for most metastases, diagnosis is not difficult according to clinical and imaging characteristics.^[3]^

Sarcoidosis is a systemic disorder of unknown etiology with a wide variety of clinical and radiological manifestations. Pathologically, the disease is characterized by presence of widespread non-caseating granulomas.^[5]^ Although, the clinical manifestations of sarcoidosis are widespread, but the lung and intrathoracic lymph nodes are almost universally affected. The most frequent radiological abnormality involves enlarged bilateral hilar and right paratracheal lymph nodes.^[6,7]^ Hodgkin’s lymphoma is a unique type of lymphoma, which is a systemic disease. In the early stage, Hodgkin’s lymphoma is mainly characterized by enlarged lymph nodes with homogeneous enhancement, especially bilateral hilar or mediastinal enlarged lymph node, which are easily confused with sarcoidosis. And then, parenchymal presentations of Hodgkin’s lymphoma and sarcoidosis are similar and indistinguishable as well.^[8]^ Moreover, patients with Hodgkin’s disease also demonstrate a decreased CD41/CD8 ratio in peripheral blood, which occasionally are found in sarcoidosis patients. The last interesting thing is that the age at onset of both also overlaps. As such, the mediastinal enlarged lymph nodes caused by Hodgkin’s lymphoma can be confused with sarcoidosis upon both clinical and imaging information.^[9]^ As is known to all, early and accurate diagnosis of Hodgkin’s lymphoma and sarcoidosis is critical because they require different therapeutic approaches.^[10]^ At present, there is no ideal imaging method and neither functional imaging nor anatomical imaging can accurately characterize lymph nodes. CT and magnetic resonance imaging are the most widely used modalities for evaluation of mediastinal enlarged lymph nodes but both have low sensitivity and specificity in differentiating between Hodgkin’s lymphoma and sarcoidosis.^[11]^ Patients with sarcoidosis and Hodgkin’s lymphoma may undergo invasive diagnostic procedures including transbronchial needle aspirate, transthoracic needle aspirate and endobronchial ultrasound guided, which may lead to some complications.^[12–15]^ There is therefore a need for noninvasive method that can accurately differentiate between sarcoidosis and Hodgkin’s lymphoma.

In recent years, quantitative spectral CT imaging has become a field of intense study. Spectral CT imaging with material decomposition reconstruction has enabled the transformation of conventional attenuation data into effective material densities, such as those of iodine and water.^[16]^ This procedure theoretically allows 2 types of tumor to be differentiated on the basis of tumor iodine density after administering an iodine contrast agent because the amount of neovascularity is unequal.^[17]^ This imaging method has been used in several clinical applications, including differentiating mediastinal lymphoma and thymoma.^[18]^ To the best of authors’ knowledge, a detailed comparison between sarcoidosis and Hodgkin’s lymphoma regarding quantitative analysis of mediastinal enlarged lymph nodes on spectral CT scan has not yet been reported.

The purpose of this study was to investigate the value of CT spectral imaging in differentiating sarcoidosis from Hodgkin’s lymphoma based on mediastinal enlarged lymph node.

## 2. Materials and methods

### 2.1. Patients and setting

This retrospective study was approved by Tangshan People’s Hospital Institutional Ethics Committee and all patients provided written informed consent. From January 2018 to August 2020, 21 patients [9 males, 12 females; median age 41.2 (27–65 years)] with untreated sarcoidosis and 39 patients [18 males, 21 females; median age 37.8 (17–62 years)] with untreated Hodgkin’s lymphoma who had undergone non-enhanced CT and dual-phase contrast-enhanced CT in dual-energy spectral mode were enrolled at the CT department, Tangshan People’s Hospital; all the patients were confirmed histologically by bronchoscopic biopsy, surgical biopsy or transthoracic needle aspiration biopsy.

### 2.2. CT examination

All patients underwent chest contrast-enhanced CT examination craniocaudally in the supine position on GE Discovery CT750 HD spiral CT, including unenhanced and biphasic enhanced scanning. Unenhanced scanning was performed in conventional helical mode and the main scanning parameters were tube voltage 120 kVp, helical pitch 1.375, smart tube current 100 to 500 mA and rotation time 0.7 seconds, respectively. And then, 80 to 100 mL nonionic contrast medium (350 mg iodine mL^−1^) was injected using a high-pressure injector via antecubital venous at an injection rate of 3.5 mL/second during enhanced scanning, which includes arterial phase (AP) and venous phase (VP). The AP scan started at 25 second after injection of contrast agent, and then the VP scan was performed at an interval of 30 second. The biphasic enhanced scanning was performed in spectral imaging mode. During a single rotation, the fast tube voltage on adjacent views was switched between 80 and 140 kVp. The main scanning parameters were tube current 275 mA, helical pitch 1.375, collimation thickness 1.25 mm, rotation speed 0.7 second and reconstructional standard 30% adaptive statistical iterative reconstruction.

### 2.3. Criteria and anatomic distribution of mediastinal enlarged lymph nodes

The criterion for significant enlargement of mediastinal lymph nodes was considered to be a short axis dimension ≥10 mm for all zones except for subcarinal lymph nodes (zone 7) were a measurement ≥12 mm was used. For axillary, internal mammary, peridiaphragmatic and retrocrural lymph nodes, short axis cut off points ≥10 mm, 5 mm, 5 mm and 6 mm were used respectively. According to Association for the Study of Lung Cancer,^[19]^ mediastinal lymph nodes were divided into 10 zones. The aforementioned zones were as follows: 1 (low cervical, supraclavicular and sternal notch), 2R (right upper paratracheal), 2L (left upper paratracheal), 3 (prevascular and retrotracheal), 4R (right lower paratracheal), 4L (left lower paratracheal), 5 (subaortic), 6 (paraaortic), 7 (subcarinal), 8 (paraesophageal), 9 (pulmonary ligament), 10R (right hilar), and 10L (left hilar).

### 2.4. Quantitative analysis

All the measurements were performed on an advanced workstation (AW4.6, Discovery CT 750 HD, GE Healthcare) with the gemstone spectral imaging viewer. Circular regions of interests (ROI) with an area of approximately 20 mm^2^ were marked on the enlarged lymph node and aorta, with a default of 70 keV for monochromatic images. The ROIs encompassed as much of the high-enhancing areas of the enlarged lymph node as possible. Areas of focal change, such as necrosis, calcification, and large vessels, were carefully avoided. To ensure consistency, all measurements were performed 3 times at different image levels, and the average values were calculated. For all measurements, the size, shape and position of the ROIs were kept consistent between the phases by applying the copy-paste function. The gemstone spectral imaging viewer software package automatically calculated the monochromatic CT values and iodine (water) concentrations for the enlarged lymph node and aorta. Two recently introduced parameters were derived from the iodine concentration (IC) measurements and monochromatic images: NIC, calculated as NIC = IC enlarged lymph node/IC aorta, ICs in the lesions were normalized to those of the aorta in order to minimize variations in patients; HU curve slop (λ_HU_), calculated as λ_HU_ = (CT value on 40 keV-CT value on 140 keV)/100.

### 2.5. Qualitative analysis

Two radiologists who were blinded to the diagnosis of the lesion qualitatively reviewed the CT images in consensus at a workstation. For each patient, characteristics of the enlarged lymph nodes were recorded, including anatomic distribution, border, coalescence, calcification, necrosis, compression, enhancement patterns and degree on 70 keV monochromatic images. The enhancement degree was described as mild-moderate or moderate-severe when the CT value increased by <25 hounsfield units (HU) or >25 HU, respectively. The changes in enhancement pattern between the phases were characterized as gradualness or persistent. Gradualness was defined as a change from low attenuation during the AP to high attenuation during the VP, and persistent was defined as the high attenuation during the AP already and the attenuation during the VP is similar to AP.

### 2.6. Statistical analysis

The datas were analyzed using SPSS v. 22.0. The 2-sample *t* test was performed to compare the quantitative parameters of normalized iodine concentrations (NICs), λ_HU_, and the monochromatic CT values between sarcoidosis and Hodgkin’s lymphoma, The differences in anatomical distribution and morphological patterns of mediastinal lymph nodes between sarcoidosis and Hodgkin’s lymphoma were compared with a chi-square test, *P* < .05 was considered statistically significant. The sensitivity (correct diagnosis of Hodgkin’s lymphomas) and specificity (correct diagnosis of sarcoidosis) of the individual phase were evaluated. The alternative hypothesis was the area under the receiver operating characteristic curve > 0.5.

## 3. Results

### 3.1. Quantitative image analysis

**Figure 1.**
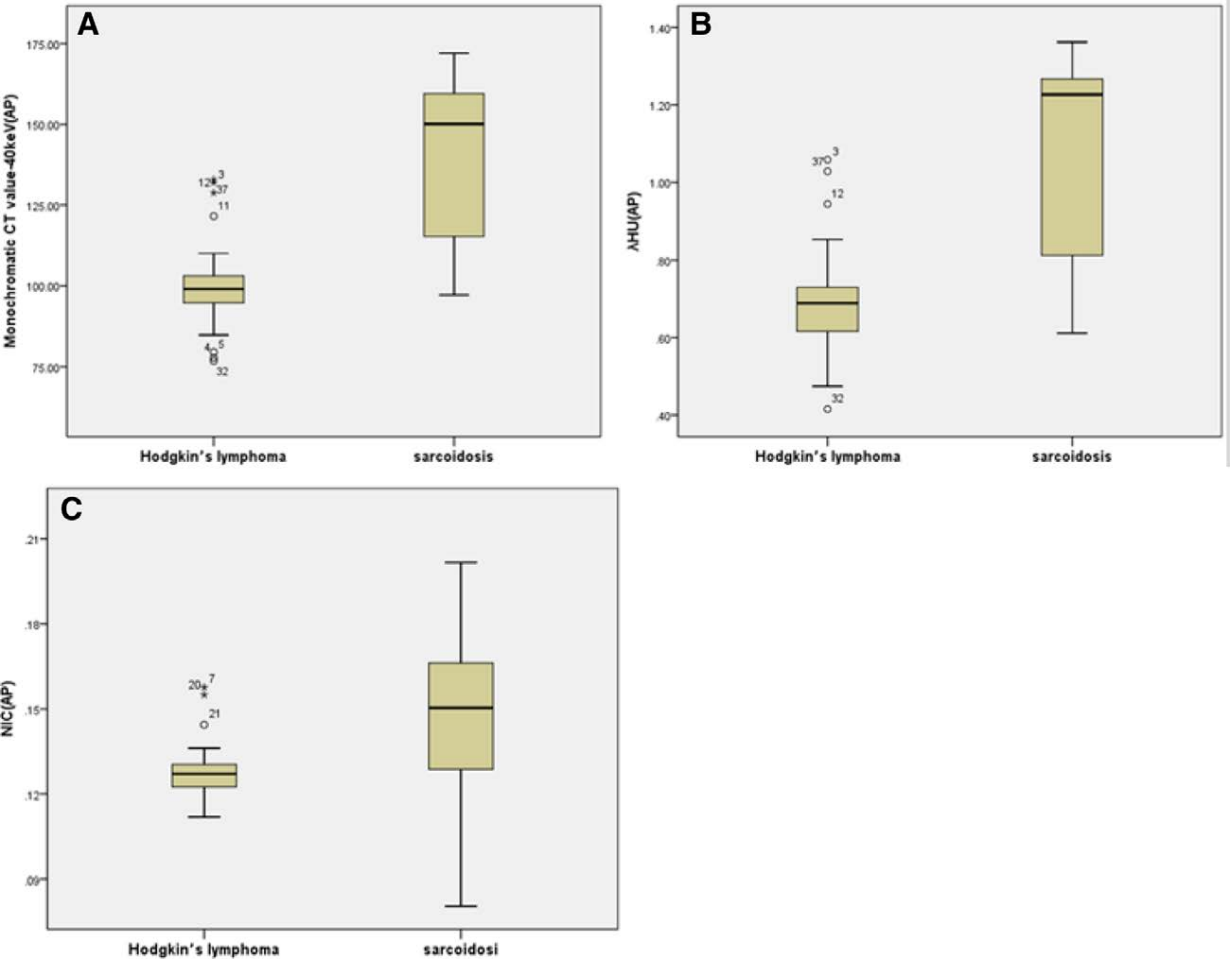
Stem-and leaf plots of (A) the monochromatic CT value on 40 keV in AP, (B) λ_HU_ during AP and (C) NIC during AP for sarcoidosis and Hodgkin’s lymphoma. AP = arterial phase, λ_HU_ = HU curve slop, NIC = normalized iodine concentration.

**Figure 2.**
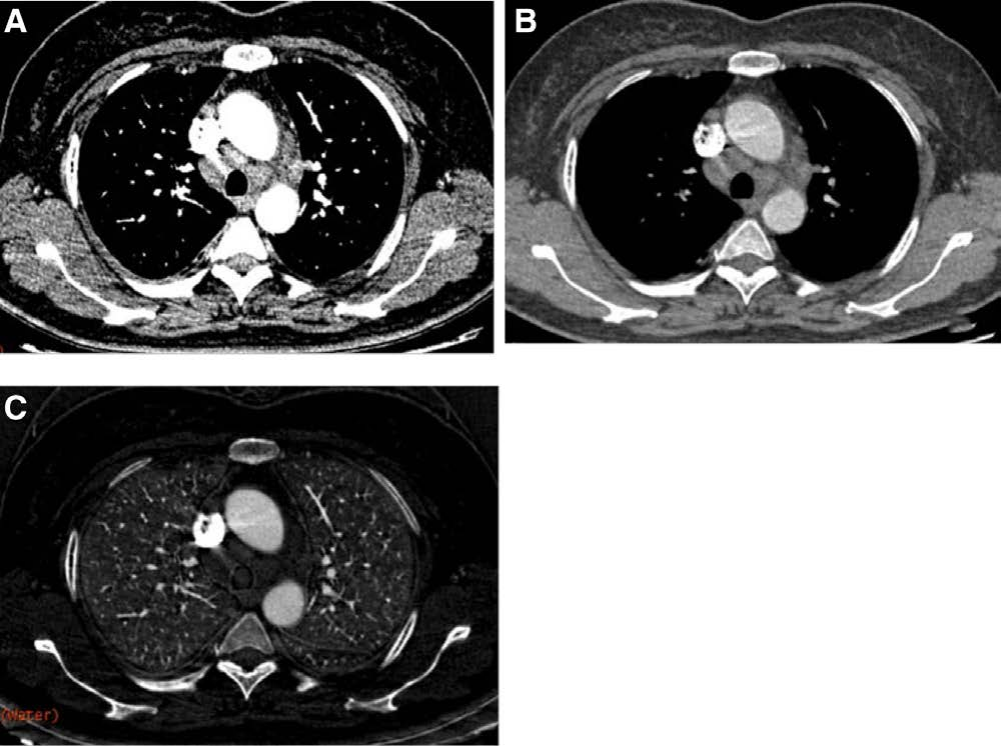
Transverse (a) monochromatic CT image obtained at 40-keV energy level a and (b) monochromatic CT image obtained at 100-keV energy level and (c) iodine-based material decomposition images obtained from spectral CT acquisition in 54-year-old female with sarcoidosis.

**Figure 3.**
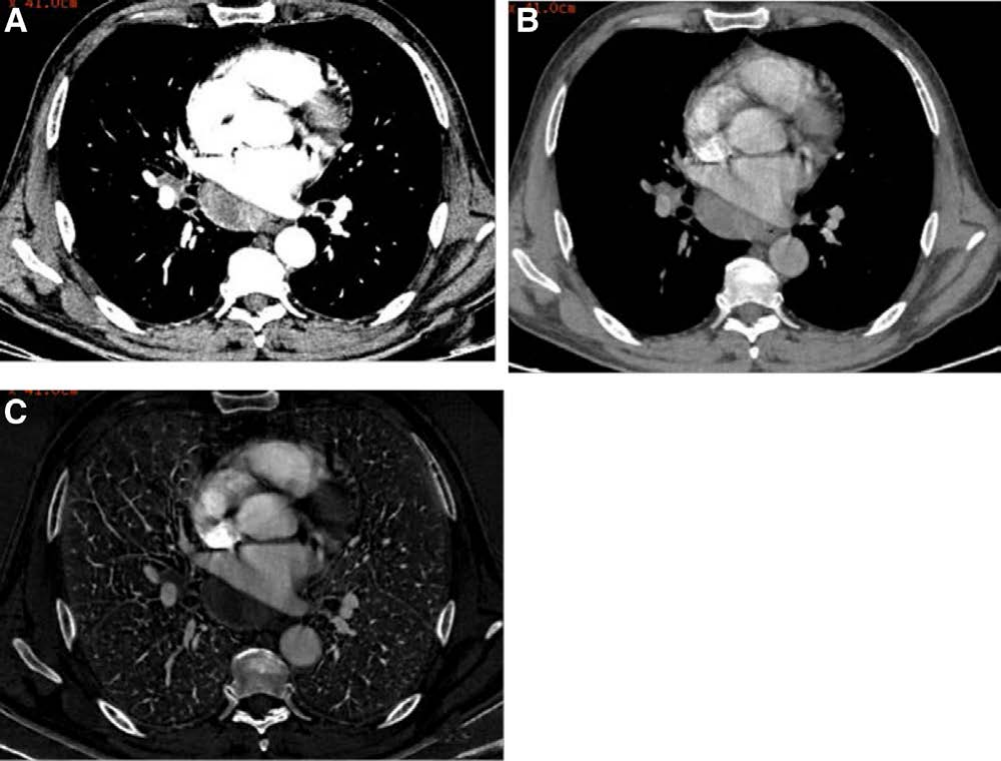
Transverse (a) monochromatic CT image obtained at 40-keV energy level and (b) monochromatic CT image obtained at 100-keV energy level and (c) iodine-based material decomposition images obtained from spectral CT acquisition in 54-year-old male with Hodgkin’s lymphoma.

Values for the quantitative parameters measured in the patients with Hodgkin’s lymphoma and in those with sarcoidosis are compared in Table 1. The CT values on 40 to 100 keV monochromatic images during the AP, the CT values on 40 to 50 keV monochromatic images during the VP, NICs in AP, λ_HU_-AP and λ_HU_-VP were all lower in Hodgkin’s lymphoma than those in sarcoidosis, and the differences were all statistically significant (*P* < .05), especially. The monochromatic CT value on 40 keV in AP (mean CT value, 99.54 ± 13.02 HU vs 142.135 ± 25.65 HU; *P* = .000) (Fig. 1a); The NICs during the AP (mean NIC, 0.13 ± 0.01 mg mL^ − 1^ vs 0.15 ± 0.03 mg mL^−1^; *P* = .011) (Fig. 1b); The λ_HU_ during the AP (mean λ_HU_-AP, 0.69 ± 0.13 vs 1.09 ± 0.27, *P* = .000) (Fig. 1c). Two example set of images in a patient with sarcoidosis is shown in Figure 2 and a patient with Hodgkin’s lymphoma in Figure 3.

**Table 1:** Quantitative assessment of sarcoidosis and Hodgkin’s lymphoma based on mediastinal enlarged lymph node at CT spectral imaging.

Parameter	Mediastinal enlarged lymph node of Hodgkin’s lymphoma	Mediastinal enlarged lymph node of sarcoidosis	*t* value	*P* value
Water concentration(AP)	1028.43 ± 6.40	1032.03 ± 8.73	−1.827	.073
Monochromatic CT value-40 keV(AP)	99.54 ± 13.02	142.13 ± 25.65	−7.131	.000
Monochromatic CT value-50 keV(AP)	76.14 ± 11.88	104.51 ± 15.18	−7.993	.000
Monochromatic CT value-60 keV(AP)	65.36 ± 9.41	79.37 ± 12.45	−4.511	.000
Monochromatic CT value-70 keV(AP)	56.56 ± 6.57	61.35 ± 7.40	−2.576	.013
Monochromatic CT value-80 keV(AP)	45.65 ± 5.59	51.30 ± 5.80	−3.684	.001
Monochromatic CT value-90 keV(AP)	41.04 ± 4.54	45.30 ± 4.28	−3.531	.001
Monochromatic CT value-100 keV(AP)	37.53 ± 3.82	41.15 ± 4.19	−3.378	.001
Monochromatic CT value-110 keV(AP)	36.39 ± 3.76	37.48 ± 3.71	−1.079	.285
Monochromatic CT value-120 keV(AP)	34.33 ± 3.65	36.04 ± 4.89	−1.528	.132
Monochromatic CT value-130 keV(AP)	32.31 ± 3.91	34.49 ± 4.86	−1.895	.063
Monochromatic CT value-140 keV(AP)	31.04 ± 3.89	32.96 ± 5.36	−1.596	.116
NIC (AP)	0.13 ± 0.01	0.15 ± 0.03	−2.784	.011
λ_HU_ (AP)	0.69 ± 0.13	1.09 ± 0.27	−7.875	.000
Water concentration (VP)	1032.06 ± 5.61	1036.11 ± 13.28	−1.657	.103
Monochromatic CT value-40 keV(VP)	153.29 ± 9.89	162.47 ± 16.02	−2.392	.024
Monochromatic CT value-50 keV(VP)	111.71 ± 7.09	117.89 ± 11.87	−2.185	.037
Monochromatic CT value-60 keV(VP)	89.77 ± 6.47	88.01 ± 9.24	0.778	.442
Monochromatic CT value-70 keV(VP)	72.99 ± 4.84	72.29 ± 6.74	0.420	.667
Monochromatic CT value-80 keV(VP)	62.89 ± 4.41	64.09 ± 5.45	−0.923	.360
Monochromatic CT value-90 keV(VP)	56.78 ± 4.38	57.83 ± 4.99	−0.849	.399
Monochromatic CT value-100 keV(VP)	51.65 ± 4.35	52.09 ± 5.49	−0.337	.737
Monochromatic CT value-110 keV(VP)	48.41 ± 4.34	48.83 ± 5.16	−0.339	.736
Monochromatic CT value-120 keV(VP)	45.51 ± 4.39	46.40 ± 5.12	−0.710	.481
Monochromatic CT value-130 keV(VP)	43.44 ± 4.31	44.45 ± 5.15	−0.803	.425
Monochromatic CT value-140 keV(VP)	41.69 ± 4.27	42.97 ± 5.10	−1.031	.307
NIC (VP)	0.40 ± 0.06	0.43 ± 0.07	−1.763	.083
λ_HU_ (VP)	1.11 ± 0.09	1.19 ± 0.14	−2.532	.014

With exception of *t* and *P* values, data are mean values ± standard deviations.

AP = arterial phase, λ_HU_ = HU curve slop, NIC = normalized iodine concentrations, VP = venous phase.

**Figure 4.**
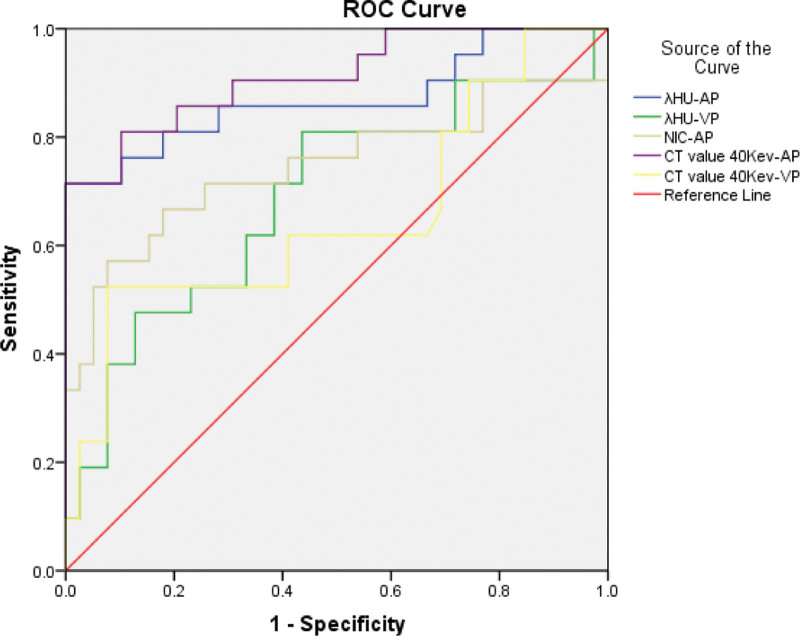
ROC curves for the monochromatic CT value on 40 keV in AP (and VP), NIC during the AP and λ_HU_ during the AP (and VP) in differentiating sarcoidosis from Hodgkin’s lymphoma based on mediastinal enlarged lymph node. AP = arterial phase, λ_HU_ = HU curve slop, NIC = normalized iodine concentration, ROC = receiver operating characteristic curve, VP = venous phase.

The area under the receiver operating characteristic curves for all parameters (Fig. 4) can be used to differentiate between sarcoidosis and Hodgkin’s lymphoma, especially the curve of the monochromatic CT value on 40 keV during the AP (0.912).

By using the receiver operating characteristic curve, we determined the parameter threshold values required to optimize both the sensitivity and specificity for differentiating between sarcoidosis and Hodgkin’s lymphoma (Table 2). For example, during the AP, a threshold monochromatic CT value on 40 keV of 139.89 would yield a sensitivity and specificity of 71.4% (28 of 39 Hodgkin’s lymphoma) and 100% (21 of 21 sarcoidosis), respectively. However, during the VP, the thresholds λ_HU_ of 1.11 would increase the sensitivity to 81.0% (32 of 39 Hodgkin’s lymphomas) but decrease the specificity to 56.4% (12 of 21 sarcoidosis).

**Table 2:** Thresholds, sensitivities, and specificities for distinguishing sarcoidosis from Hodgkin’s lymphoma based on mediastinal enlarged lymph node.

Parameter	AUC	Threshold	Sensitivity	Specificity
CT value-40 keV-AP	0.912	139.89	71.4% (28)	100% (21)
CT value-40 keV-VP	0.653	167.26	52.4% (20)	92.3% (19)
NIC-AP	0.746	0.14	57.1% (23)	92.3% (19)
λ_HU_-AP	0.871	1.10	71.4% (28)	98.7% (20)
λ_HU_-VP	0.689	1.11	81.0% (32)	56.4% (12)

Sensitivity values are cited as percentages. Data in parentheses are numbers of Hodgkin’s lymphoma (n = 39) used to calculate percentages. Specificity values are cited as percentages. Data in parentheses are numbers of sarcoidosis (n = 21) used to calculate percentages.

AP = arterial phase, AUC = area under the curve, λHU = HU curve slop, NICs = normalized iodine concentrations, VP = venous phase.

### 3.2. Qualitative image analysis

The results of the qualitative analysis of 70 keV monochromatic images are provided in Table 3. The anatomic distribution, coalescence, calcification, compression, enhancement patterns and degree differed between sarcoidosis and Hodgkin’s lymphoma based on mediastinal enlarged lymph node (*P* < .05). The border and necrosis between the 2 groups were similar (*P* > .05).

**Table 3:** Qualitative CT assessment of mediastinal enlarged lymph node between 2 groups.

CT signs		Sarcoidosis (21)		Hodgkin’s Lymphoma (39)		*χ*^2^ value	*P* value
		n	%	n	%		
Anatomic distribution	1	7	33.3	25	64.1	5.192	.023
	2	16	76.2	30	76.9	0.004	.949
	3	8	38.1	28	71.8	6.459	.011
	4	19	90.5	31	79.5	1.569	.210
	5	15	71.4	25	64.1	0.330	.566
	6	11	52.4	21	53.8	0.012	.914
	7	16	76.2	28	71.8	0.135	.713
	8	7	33.3	12	30.8	0.041	.839
	9	3	14.2	4	10.3	0.215	.643
	10*	19	90.5	26	66.7	4.127	.042
	10R	2	9.5	8	20.5	1.187	.276
	10L	0	0.0	8	20.5	4.970	.026
	10B	17	81.0	10	25.7	16.873	.000
	Axillary	1	4.8	11	28.2	4.689	.030
	Peridiaphracgmatic	1	4.8	9	23.1	3.297	.069
	Internal mammary	2	9.5	11	28.2	2.807	.094
	Retrocrural	0	0.0	6	15.4	3.590	.058
Border	Clear	20	95.2	35	89.7	0.539	.463
	Fuzzy	1	4.8	4	10.3		
Coalescence	Yes	1	4.8	29	74.4	26.447	.000
	No	20	95.2	10	25.6		
Calcification	Yes	5	23.8	1	2.6	6.846	.009
	No	16	76.2	38	97.4		
Necrosis	Yes	0	0	3	7.7	1.700	.192
	No	21	100	36	92.3		
compression	Yes	1	4.8	19	48.7	11.868	.001
	No	20	95.2	20	51.3		
Enhancement pattern	Mild-moderate	5	23.8	27	69.2	11.315	.001
	Moderate-severe	16	76.2	12	30.8		
Enhancement degree	Gradualness	2	9.5	30	76.9	24.914	.000
	Persistent	19	90.5	9	23.1		

* 10R (Right unilateral hilar), 10L (Left unilateral hilar), 10B (Bilateral hilar); according to the International Association for the Study of Lung Cancer lymph node map.

## 4. Discussion

Sarcoidosis is one of the most important lung diseases, mainly manifested as mediastinal lymph node enlargement, with a typical pattern of involvement, including the bilateral hilar and right paratracheal zones.^[20]^ However, this mode of participation is not specific and can be found in Hodgkin’s lymphoma, especially the nodularsclerosis type.^[21,22]^ In addition, parenchymal presentations and the age at onset of both also overlap. Moreover, the treatments of sarcoidosis and Hodgkin’s lymphoma are very different. Therefore, it is of great significance to correctly differentiate lymphoma from sarcoidosis before operation.

CT is the most preferred imaging method for assessing sarcoidosis and can help differential diagnosis of sarcoidosis and other mediastinal abnormalities based on mediastinal enlarged lymph node.^[23]^ Previous study^[11]^ has discussed the important role of CT in the diagnosis of sarcoidosis and Hodgkin’s lymphoma based on mediastinal enlarged lymph node. However, it mainly focused on the qualitative analysis of the imaging characteristics of enlarged lymph nodes, rather than quantitative analysis.

The photon-integrating detector was used by traditional X-ray CT system to collect photons in the entire X-ray spectrum, ignoring the spectral response of the material. Therefore, for biological soft tissues, conventional CT usually does not have high enough contrast resolution.^[24–26]^ But the monochromatic images generated by spectral CT describes the appearance of the imaging object if the X-ray source only generates single energy X-ray photons. The attenuation of different tissues varies with the change of X-ray energy. Lower monochromatic energy can improve the image density resolution and help to display the lesions. According to our study results, based on mediastinal enlarged lymph node, the CT values on 40 to 100 keV monochromatic images in AP and 40 to 50 keV in VP were higher in sarcoidosis than those in Hodgkin’s lymphoma, which confirms that lower monochromatic energy can improve contrast resolution for lesions.

The IC of the lesions obtained from the decomposition images of iodine-based materials is quantitative, so it may be a useful parameter.^[24]^ NIC is the indirect reflection of the iodine content in the lesion, which reflects enhancement degree of lesions. Our study shows that based on mediastinal enlarged lymph node, the NIC was higher in sarcoidosis than Hodgkin’s lymphoma during the AP. However, no significant differences in NIC was observed in the VP. Our fingdings indicate that the blood supply (measured by contrast agent concentration) of Hodgkin’s lymphoma in the AP is lowerer than that of sarcoidosis. Histologically, sarcoidosis is characterized by non-caseous granulomas, which are composed of epithelioid cells with uniform size and morphology surrounded by fibroblasts and gelatins. In addition, sarcoidosis is easy to form perivascular granulomatous reaction, which aggregates a lot of inflammatory cells. Due to histopathological features, there is a higher IC during the AP and persistent in VP. Histologically, highly proliferative lymphocyte precursors were contained in the enlarged lymph nodes for Hodgkin’s lymphoma. In addition, treatment-induced necrosis will occur which may be associated with a good prognosis^[27]^; Therefore, the contrast agent slightly enters the tumor vessels in AP phase and reaches the peak in VP phase.

This study demonstrates that there was a significant difference in the slope of the spectral curve between Hodgkin’s lymphoma and sarcoidosis based on mediastinal enlarged lymph node in both AP and VP. The material CT value varying with X-ray energy and the absorption characteristics of X-ray with different energy are reflected by the spectral curve. Different substances show changes in the structure of chemical molecules, and energy attenuation curves have been modified by various chemical molecules.^[28]^ Thus, the chemical composition of substances can be distinguished by comparing the slopes of the spectrum curves.^[29]^ Sarcoidosis is characterized by non-caseous granulomas and is composed of epithelioid cells, but the lymphomatous lymph nodes are mainly composed lymphocytes and RS cells. Therefore, different absorption characteristics could be observed between Hodgkin’s lymphoma and sarcoidosis because of different histological structures.

The receiver operating characteristic curve curves analysis in our study showed that the monochromatic CT value on 40 keV in AP and λ_HU_ in VP (139.89 Hu and 1.11, respectively) had the best sensitivities and specificities of 81.0% and 100% respectively in distinguishing sarcoidosis and Hodgkin’s lymphoma based on mediastinal enlarged lymph node. However, the accuracy of these thresholds evaluated needs to be further confirmed by using a larger sample size in future studies, because there is only a specific population in our study.

There are two main limitations in our study. On the one hand, the results in our study were still in the preliminary stage and thus need to be verified in future studies with a larger sample size due to small sample size. On the other hand, the different stages of sarcoidosis and the different subtypes and pathological grading of Hodgkin’s lymphoma were not compared in this study,^[30]^ and the sample size needs to be further expanded for further research.

In conclusion, the CT spectral imaging can decompose materials and analyze some additional quantitative CT imaging parameters such as monochromatic CT value, spectral curve slope and NIC, which is helpful to improve the accuracy of distinguishing sarcoidosis and Hodgkin’s lymphoma based on mediastinal lymph node enlargement, so as to instruct the clinical treatment better.

## Acknowledgments

We thank all of the patients enrolled for this study.

## Author contributions

**Conceptualization:** Yongliang Liu.

**Investigation:** Huijing Wu.

**Writing – review & editing:** Lixiu Cao.
